# Real-world disease burden, patient journey and treatment patterns in eosinophilic granulomatosis with polyangiitis in Europe and the USA

**DOI:** 10.1016/j.ero.2026.03.016

**Published:** 2026-04-22

**Authors:** Robert Spiera, Paul Dolin, Bo Ding, Priya Jain, Lotte Westerink, Chris Edmonds, Tia Pennant, Oliver-Thomas Carter, Fritha Hennessy, Stephanie Yanjing Chen

**Affiliations:** 1 Scleroderma, Vasculitis, and Myositis Center, Hospital for Special Surgery, Weill Cornell Medical College, NY, USA; 2 BioPharmaceuticals Medical, AstraZeneca, Cambridge, UK; 3 BioPharmaceuticals Medical, AstraZeneca, Gothenburg, Sweden; 4 Health Economics & Payer Evidence, AstraZeneca, Cambridge, UK; 5 Market Access and Pricing, AstraZeneca, Gaithersburg, MD, USA; 6 Adelphi Real World, Bollington, UK; 7 BioPharmaceuticals Medical, AstraZeneca, Gaithersburg, MD, USA

## Abstract

**Objectives:**

An enhanced understanding of eosinophilic granulomatosis with polyangiitis (EGPA) in clinical practice may help identify areas where patient management could be improved. The objectives of this study were to examine the real-world demographics, patient diagnostic journey, disease burden, treatment patterns, and health-related quality of life (HRQoL) of patients with EGPA.

**Methods:**

Data were drawn from the Adelphi Real World EGPA Disease Specific Programme, a cross-sectional survey of patients with EGPA and their physicians in Europe (France, Germany, Italy, Spain, and the UK) and the USA from July to December 2023.

**Results:**

The study included 121 physicians and 503 patients. Most patients were White (89%), the mean (SD) age was 49.5 (15.3) years, and the distribution of sexes was balanced. Mean (SD) time between sign/symptom onset and EGPA diagnosis was 9.8 (19.1) months. Patients had a mean (SD) of 5.5 (3.8) signs and/or symptoms at diagnosis, and physician-perceived severity of EGPA was mild in 20%, moderate in 55%, and severe in 24% of patients. Glucocorticoids were the most prescribed therapies (79%), and the use of interleukin-5-/receptor alpha-targeted therapies was low (21% mepolizumab, 7% benralizumab, <1% reslizumab). Patient-reported HRQoL and work productivity were most impacted in those with organ damage, oral glucocorticoid dose ≥10 mg/day, blood eosinophil count ≥300 cells/μL, or relapse, refractory, deteriorating, moderate, or severe disease.

**Conclusions:**

EGPA is associated with a considerable disease burden. Increased disease awareness to facilitate prompt diagnosis and treatment and optimised management to achieve remission and enhance patients’ HRQoL are needed.

**WHAT IS ALREADY KNOWN ON THIS TOPIC**

• Patients with eosinophilic granulomatosis with polyangiitis (EGPA) experience a considerable disease burden, and the diverse clinical manifestations of EGPA can require multiple specialist visits and referrals, which result in delayed diagnosis and risk of organ damage.
• Treatment of EGPA typically involves long-term high-dose glucocorticoids and/or other immunosuppressant use, which are associated with severe adverse effects.

**WHAT THIS STUDY ADDS**

• This study provides real-world data on patients with EGPA in Europe and the USA.
• There is a broad range of timeframes for patient journeys to EGPA diagnosis, and patients have a high disease burden by the time of diagnosis.
• There is often discordance between physician and patient perspectives in terms of disease severity and signs/symptoms reported.
• Despite the availability of targeted therapies, there is an ongoing reliance on glucocorticoids for treatment.

**HOW THIS STUDY MIGHT AFFECT RESEARCH, PRACTICE OR POLICY**

• The observation of diagnostic delays in this study should encourage a call to action to increase awareness of EGPA and be used to help drive practice changes to facilitate quicker patient identification, referral, and diagnosis pathways.
• Knowledge of the discordance between physician and patient perspectives could help physicians improve their understanding of and communication with their patients to ensure better treatment outcomes.
• The data from this study support the continued need for optimised management and glucocorticoid-sparing, targeted therapies in practice to achieve remission and enhance patients’ health-related quality of life.

Alt-text: Unlabelled box dummy alt text

## INTRODUCTION

Eosinophilic granulomatosis with polyangiitis (EGPA) is a rare disorder characterised by vasculitis of small-to-medium blood vessels, blood and tissue eosinophilia, asthma, and granulomatous inflammation affecting multiple organs [[1], [2], [3], [4]]. The estimated global prevalence of EGPA is 34.44 cases per 1,000,000 people, and global incidence is approximately 2.15 cases per 1,000,000 person-years [5]. The associated disease burden of EGPA is high, with patients experiencing multiorgan damage and dysfunction, relapses, and concomitant conditions (often considered as components of EGPA), which in turn contribute to high healthcare resource utilisation (HCRU) [3,4,6]. Relapses, asthma flares, and organ damage may also have a substantial negative impact on patients’ health-related quality of life (HRQoL) [7].

EGPA is classified as an antineutrophil cytoplasmic antibody (ANCA)-associated vasculitis, with detectable ANCAs in approximately 40% of cases [4]. The clinical manifestations of EGPA are heterogenous, and the frequency of certain organ manifestations often differs according to ANCA status. Features driven by eosinophilic inflammation, such as cardiomyopathy and gastroenteritis, are more frequent in ANCA-negative patients, while glomerulonephritis and vasculitis are more frequent in ANCA-positive patients [4]. The diagnosis of EGPA can be challenging and often delayed due to the multiorgan nature of the disease, as well as its rarity and overlap with other disorders [2,4]. Given its complexity, a multidisciplinary approach for diagnosis and treatment is usually required [8].

Treatment of EGPA typically includes oral glucocorticoids, which may initially be at high doses in some patients, followed by long-term exposure at lower doses [4,9]. Immunosuppressants are also used, with the aim of reducing glucocorticoid use, inducing and maintaining remission, and managing relapses [1,2,4,9]. Glucocorticoids and many immunosuppressants are associated with severe adverse effects such as osteoporosis, cardiometabolic disease, elevated risk of serious infections, gastrointestinal, dermatologic and neuropsychiatric disorders, and impaired fertility [4,8,10]. More recently, novel biologics such as the anti-interleukin-5 (IL-5)/receptor alpha (Rα) therapies mepolizumab and benralizumab have demonstrated efficacy in inducing and maintaining remission in patients with relapsing/refractory EGPA [11]. The use of biologics for the maintenance treatment of EGPA is now recommended in guidelines [4,9], with mepolizumab having been approved in Europe in 2021 and the USA in 2017 [2,12,13], and benralizumab having been approved in these regions in 2024 [14,15].

The evolving treatment landscape, shaped by recent EGPA guidelines and novel targeted therapies, underscores the need for continued investigation of EGPA within clinical practice. The objectives of this study were to understand the real-world demographics, patient journey, disease burden, treatment patterns, and HRQoL of patients with EGPA in Europe and the USA.

## METHODS

### Study design and study population

This study comprised a secondary analysis of data from the Adelphi Real World EGPA Disease Specific Programme (DSP), a cross-sectional survey of physicians and patients in Europe (France, Germany, Italy, Spain, and the UK) and the USA from July to December 2023. DSPs are independent, impartial data sources and are not designed to address any prespecified research questions or hypotheses. The DSP methodology has been previously described [16,17], validated [18], and demonstrated to be representative and consistent over time [19]. Data collection was undertaken in line with European Pharmaceutical Market Research Association guidelines, as all data were aggregated and deidentified before receipt. As such, the study did not require ethics committee approval. Each survey was performed in full accordance with relevant legislation at the time of data collection, including the US Health Insurance Portability and Accountability Act of 1996 [20], the European Pharmaceutical Market Research Association guidelines [21], and the Health Information Technology for Economic and Clinical Health Act legislation [22].

A representative sample of physicians was recruited to participate in the DSP by local fieldwork agents, following completion of a short screening questionnaire. Eligible physicians included rheumatologists, pulmonologists, allergists and immunologists, gastroenterologists, internal medicine specialists, dermatologists, otolaryngologists, cardiologists, neurologists, and haematologists who were responsible for the management and treatment of 2 or more patients with EGPA. Physician participation was financially incentivised, with reimbursement upon survey completion according to fair market research rates.

Participating physicians were first required to complete a physician survey focusing on their characteristics, patient caseload, and attitudes and perceptions regarding disease management and treatment alternatives. Each physician then completed patient record forms (PRFs) for 2 to 4 consecutively consulting patients with a diagnosis of EGPA. PRFs were cross-sectional; however, there was a retrospective element of data collection using physician-reported information and details from patients’ medical records. The patient information collected included: demographics; signs/symptoms, based on the Birmingham Vasculitis Activity Score (BVAS) version 3 [23]; organ damage; physician-perceived disease status, severity and expected progression; concomitant conditions; time to EGPA diagnosis; physician involvement in diagnosis and treatment; current treatment; and HCRU.

The same patients for whom physicians completed a PRF were invited to independently complete a voluntary patient self-completion (PSC) survey. Information collected included caregiver support (where applicable), symptoms and severity, and current treatment. Additionally, 3 patient-reported outcome measures were captured: EuroQol-5 Dimension 5-level (EQ-5D-5L) [24], scored from 0 (worst health) to 1 (full health); Work Productivity and Activity Impairment (WPAI), scored from 0% (no impairment) to 100% (severe impairment) [25]; and Asthma Control Questionnaire (ACQ), scored from 0 (best asthma control) to 6 (worst asthma control).

PRFs and PSCs were linked via unique, pseudoanonymised physician- and patient-coded numbers.

Eligible patients were aged ≥10 years, with a physician-confirmed diagnosis of EGPA.

### Statistical methods

Descriptive statistics were used to summarise continuous variables (means, SDs, medians, IQRs) and categorical variables (frequencies, percentages). As questions in the PRF and PSC could be skipped, each variable was described using the maximum sample available to that variable. Missing data were not imputed.

## RESULTS

### Physician study participation

A total of 121 physicians were enrolled in the survey, of whom rheumatologists (22%, n = 27/121) and pulmonologists (19%, n = 23/121) represented the largest proportions. In the 12 months prior to survey completion, the mean (SD) number of patients with EGPA seen by enrolled physicians was 26.0 (33.3), and physicians personally managed a mean (SD) of 22.4 (31.3) patients with EGPA.

### Patient demographics

Data were reported by physicians on a total of 503 patients with EGPA (France, n = 80; Germany, n = 80; Italy, n = 91; Spain, n = 75; UK, n = 57; USA, n = 120). The mean (SD) age was 49.5 (15.3) years, most patients for whom data were available were White (89%, n = 375/423), and the distribution of sexes was balanced (Table). Additional patient demographics are available in Supplementary Table S1.

**Table:** Patient demographics and clinical characteristics at the time of survey

Variable	Total patients with EGPA (N = 503)
**Patient age (years)**	**n = 503**
Mean (SD)	49.5 (15.3)
**Patient sex, n (%)**	**n = 503**
Male	253 (50)
Female	249 (50)
Intersex	1 (<1)
**Ethnicity, n (%)**^a^	**n = 423**
White	375 (89)
Black (African American, African, or Caribbean)	23 (5)
Other^b^	26 (6)
**Additional support/care required, n (%)**	**n = 503**
Yes	110 (22)
No	368 (73)
Don’t know	25 (5)
**Caregiver(s) involved, n (%)**	**n = 110**
Partner/spouse	86 (78)
Child aged ≥18 years	16 (15)
Parent/guardian	13 (12)
Other relative(s)	6 (5)
Professional caregiver(s)	4 (4)
Friend(s)/neighbour(s)	3 (3)
Other nonprofessional caregiver(s)	2 (2)
**Total number of signs and/or symptoms experienced currently, n**	**n = 503**
Mean (SD); [min, max]	2.9 (2.6); [1, 14]
**Organ damage (grouped), n (%)**^c^	**n = 503**
Yes	364 (72)
No	65 (13)
All organ damage this patient suffers with predates their EGPA	43 (9)
Don’t know	31 (6)
**Disease status, n (%)**	**n = 503**
In remission	357 (71)
In relapse	60 (12)
Refractory	48 (10)
Too early to decide	19 (4)
Other	19 (4)
**Physician-perceived severity (currently), n (%)**^d^	**n = 503**
No active disease	136 (27)
Mild	275 (55)
Moderate	73 (15)
Severe	19 (4)

EGPA, eosinophilic granulomatosis with polyangiitis.

a Information on ethnicity was not requested/provided in France.

b Includes East or Southeast Asian, South Asian, Middle Eastern, North African, American Indian, Indigenous American, Alaska Native, and South or Central American Native.

c This was any organ damage that the patient had at the survey date that either occurred or worsened since their EGPA diagnosis. The physician provided this information using the patients’ medical records. The organ damage that was available to the physicians to select were musculoskeletal, skin/mucous membranes, ocular, ears, nose and throat, pulmonary, cardiovascular, peripheral vascular disease, gastrointestinal, renal, neuropsychiatric, and/or other organs. The source of organ damage (eg, due to EGPA or therapies) was not specified on the survey.

d No definitions of mild, moderate, or severe were provided in the survey, so the definitions of severity were up to each physician’s own judgement.

In terms of patient-reported results, a total of 180 patients completed PSCs. Mean (SD) age was 48.7 (14.8) years, and the distribution of sexes was similar to that of the overall population (52% female, 48% male).

### Physician-reported results

#### Patient journey

**Figure 1:**
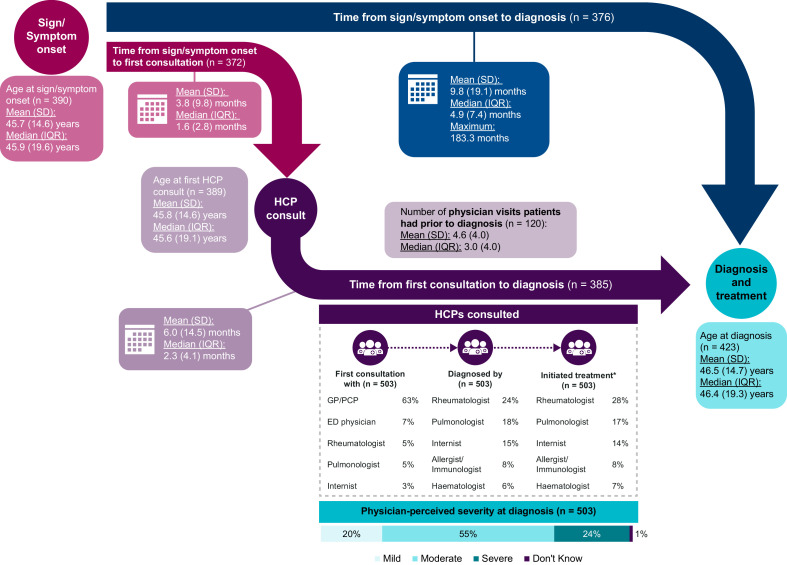
Physician-reported patient journey from onset of EGPA signs and/or symptoms to diagnosis and treatment. *Defined as the first physician to initiate treatment after the patient had a confirmed EGPA diagnosis. ED, emergency department; EGPA, eosinophilic granulomatosis with polyangiitis; GP; general practitioner; HCP, healthcare professional; PCP, primary care physician.

Physicians reported that patients (n = 372) experienced a mean (SD) of 3.8 (9.8) months between the first onset of signs/symptoms and first consultation with an HCP about EGPA. There was a mean (SD) duration of 6.0 (14.5) months between the first consultation and physician-confirmed EGPA diagnosis (n = 385). The mean (SD) time between onset of signs/symptoms and EGPA diagnosis was 9.8 (19.1) months, with a maximum of 183.3 months (n = 376) (Fig 1). The duration between signs/symptoms onset and EGPA diagnosis was more than 24 months for 8% (n = 31/376) of patients.

Initial HCP consultation was typically with a general practitioner or primary care physician (63%, n = 317/503). Rheumatologists were most likely to diagnose patients (24%, n = 120/503), followed by pulmonologists (18%, n = 89/503) and internists (15%, n = 77/503) (Fig 1). Patients (n = 456) had been in the care of their reporting physician for a mean (SD) duration of 27.9 (29.0) months. Physicians began patient management on the day of diagnosis for 45% (n = 192/423) of cases, with 39% (n = 163/423) of patients being managed by the physician after diagnosis, and 16% (n = 68/423) being managed before diagnosis (Supplementary Table S2).

#### Disease burden

Physicians reported caregiver support was required by 22% of patients, with a mean (SD) duration of 23.1 (23.6) hours of support provided each week. For patients requiring caregiver support (n = 110), this was provided by the spouse or partner in 78% (n = 86/110), their child aged ≥18 years in 15% (n = 16/110), and a parent or guardian in 12% (n = 13/110; all these patients were <18 years old) of cases (Table).

**Figure 2:**
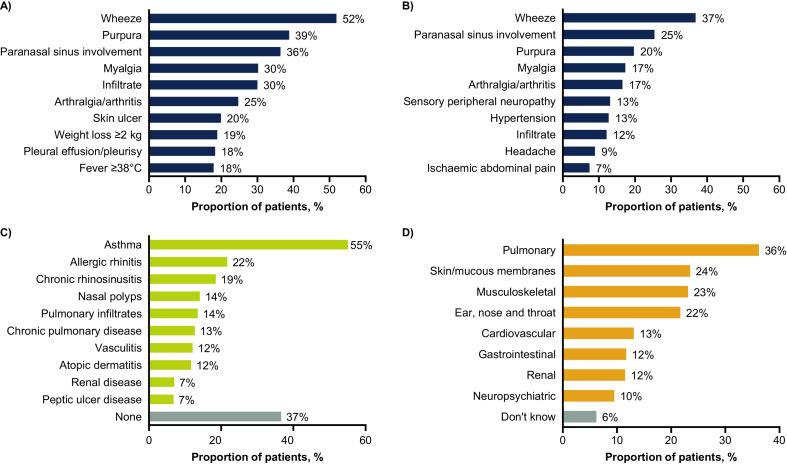
Physician-reported patient signs and symptoms, concomitant conditions and organ damage (N = 503). A, 10 most common signs and symptoms at diagnosis. B, 10 most common signs and symptoms at survey completion. C, 10 most common concomitant conditions at survey completion. D, organ damage at survey completion.

Physicians reported that patients had a mean (SD) of 5.5 (3.8) signs and/or symptoms at diagnosis and 2.9 (2.6) signs and/or symptoms at survey completion. At diagnosis, the top 3 most reported signs/symptoms were wheeze (52%, n = 261/503), purpura (39%, n = 195/503), and paranasal sinus involvement (36%, n = 183/503). At survey completion, these were wheeze (37%, n = 185/503), paranasal sinus involvement (25%, n = 128/503), and purpura (20%, n = 99/503) (Fig 2). A full list of physician-reported signs and symptoms experienced by patients at diagnosis and survey completion is reported in Supplementary Table S3.

The most common physician-reported patient concomitant condition at survey completion was asthma, which was reported in 55% (n = 277/503) of patients (Fig 2). Asthma was predominantly mild (45%, n = 126/277) or moderate (45%, n = 126/277), and 30% (n = 83/277) of patients with concomitant asthma reported having an exacerbation in the 12 months prior to survey completion. Just over one-third of patients had no concomitant conditions (Fig 2). A full list of concomitant conditions is provided in Supplementary Table S4.

Physician-determined organ damage due to EGPA primarily impacted the lungs (36%, n = 182/503), skin/mucous membranes (24%, n = 118/503), and the musculoskeletal system (23%, n = 116/503) (Fig 2).

At diagnosis, physician-perceived severity of EGPA was mild in 20% (n = 99/503), moderate in 55% (n = 277/503), and severe in 24% (n = 122/503) of patients. At survey completion, EGPA was reported as being mild in 55% (n = 275/503), moderate in 15% (n = 73/503), and severe in 4% (n = 19/503) of patients (Table; Supplementary Table S2).

#### Treatment patterns

**Figure 3:**
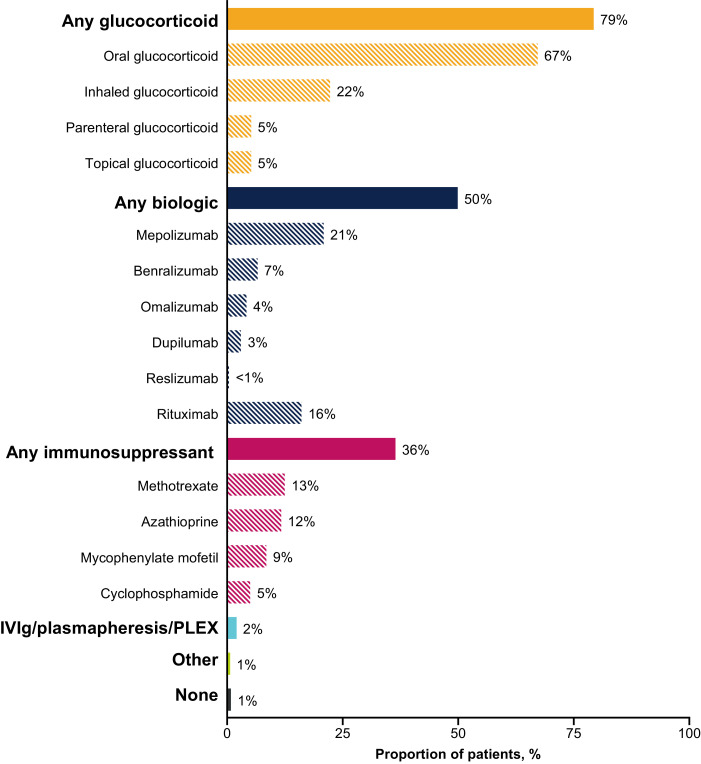
Physician-reported treatment prescriptions in patients with EGPA at survey completion (N = 503). EGPA, eosinophilic granulomatosis with polyangiitis; IVIg, intravenous immunoglobulin; PLEX, plasma exchange.

Physicians reported a high level of glucocorticoid use in patients with EGPA, with 79% (n = 399/503) of patients receiving a glucocorticoid at the time of data collection, and 85% (n = 429/503) having received glucocorticoids within their 5 most recent treatment regimens. Oral glucocorticoids were the most prescribed (67%, n = 338/503), followed by inhaled glucocorticoids (22%, n = 112/503) (Fig 3). Mean (SD) prednisone-equivalent glucocorticoid dose was 17.0 (20.4) mg/day, and the median (IQR) daily systemic dose was 8.0 (15.0) mg/day. Of patients receiving systemic glucocorticoids, 21% (n = 72/351) were receiving doses >20 mg/day (Supplementary Table S5).

Half of patients (50%, n = 251/503) were receiving treatment with a biologic at survey completion. In terms of anti-IL-5/Rα treatments, 21% (n = 105/503) of patients were prescribed mepolizumab, 7% (n = 33/503) benralizumab, and <1% (n = 2/503) reslizumab (Fig 3).

**Figure 4:**
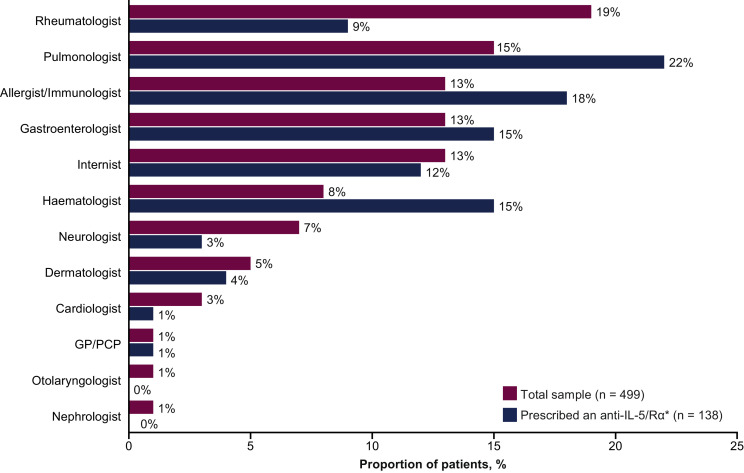
Physician-reported specialty of HCP who initiated current therapy for EGPA. *Includes those prescribed mepolizumab or benralizumab ± other treatments (anti-IL-5/Rα only [n = 34]). Anti-IL-5/Rα, anti-interleukin-5/interleukin-5 receptor alpha; EGPA, eosinophilic granulomatosis with polyangiitis; GP, general practitioner; HCP, healthcare professional; PCP, primary care physician.

Although rheumatologists were initiating treatment most frequently, they prescribed anti-IL-5/Rα treatment less frequently than many other specialists. Pulmonologists and allergists/immunologists prescribed anti-IL-5/Rα treatment most frequently (Fig 4). From a geographical perspective, anti-IL-5/Rα treatment was most prescribed by HCPs in Italy (51%, n = 46/91) and Spain (35%, n = 26/75), while prescriptions were lower in the UK (28%, n = 16/57), Germany (21%, n = 17/80), France (18%, n = 14/80), and the USA (18%, n = 21/120). See Supplementary Table S6 for treatment prescriptions stratified by country.

Patients were on treatment for a mean (SD) of 19.7 (27.7) months. Most physicians were satisfied with their patients’ treatment (88%, n = 428/489) even though approximately one-third of them thought that control of disease was suboptimal (34%, n = 164/489). The most common reason for treatment choice was symptom relief (61%, n = 306/499). Perceived areas for improvement of existing treatment were speed of onset of action (26%, n = 132/499), better tolerated side-effect profile (26%, n = 130/499), and reductions in mortality (17%, n = 85/499) (Supplementary Table S5).

Physicians reported that most patients (84%, n = 418/499) had an improvement in symptom severity after starting treatment. Improvements in wheeze (49%, n = 205/418), purpura (34%, n = 142/499), and paranasal sinus involvement (30%, n = 124/499) were most commonly reported with treatment (Supplementary Table S5).

### Patient-reported HRQoL and work productivity

At diagnosis, most patients who completed PSCs (n = 180) considered their symptoms to be severe (41%, n = 73/180) or moderate (42%, n = 76/180). At survey completion, fewer patients reported symptoms as being severe (6%, n = 11/174) or moderate (27%, n = 47/174). Most patients (83%, n = 148/178) indicated that their disease had not worsened since diagnosis. The most common symptoms reported by patients at survey completion were fatigue (52%, n = 93/179), wheeze (40%, n = 71/179), and shortness of breath (38%, n = 68/179). Most patients expressed satisfaction with their current treatment (very satisfied 26%, n = 45/174; satisfied 48%, n = 84/174).

**Figure 5:**
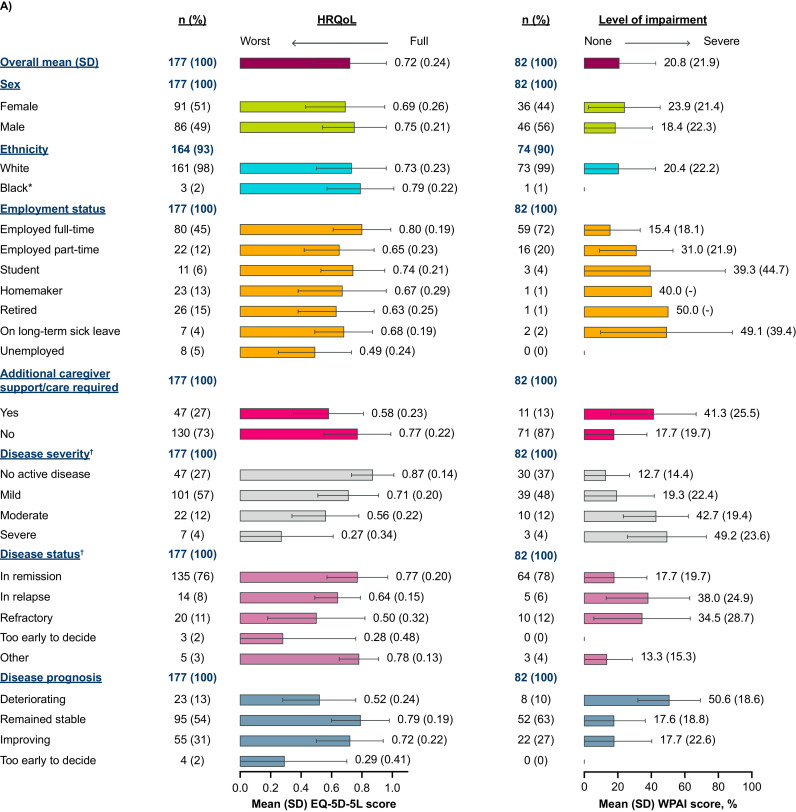

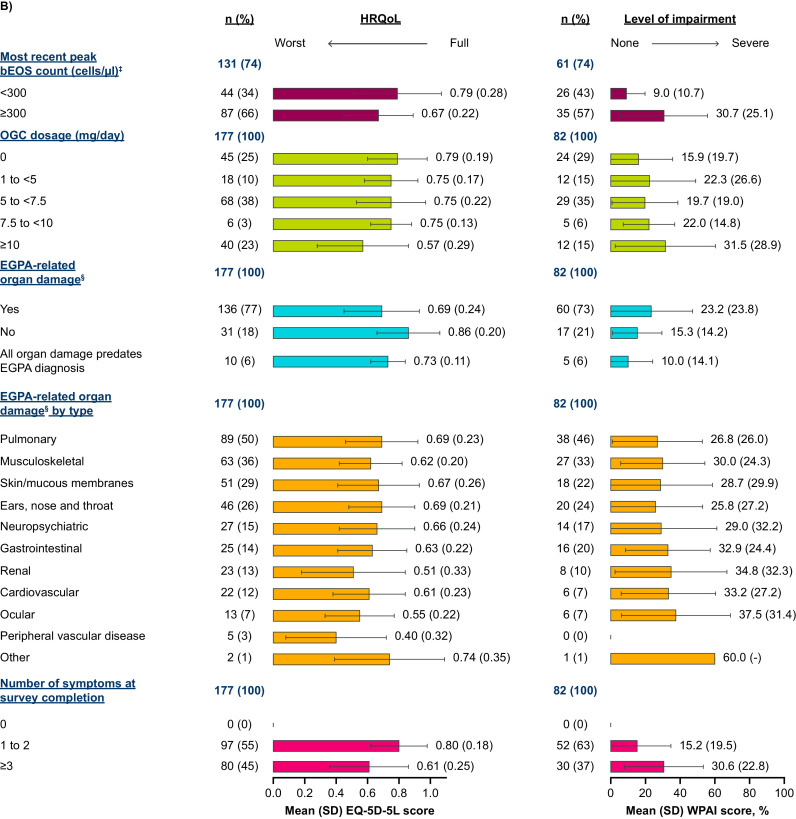
EQ-5D-5L and WPAI scores at survey completion. A, Stratified by patient demographics and physician-perceived EGPA status. B, Stratified by patient clinical characteristics. *African American, African, or Caribbean; ^†^Definitions for disease status and severity were based on physician judgement; ^‡^Time since most recent bEOS count from data collection varied between patients: 64% <3 months, 28% ≥3 to <6 months, 8% ≥6 months. Ongoing treatment may have affected peak bEOS counts; ^§^Organ damage was captured through medical records available to the physicians. bEOS, blood eosinophil; EGPA, eosinophilic granulomatosis with polyangiitis; EQ-5D-5L, EuroQoL 5-dimension 5-level questionnaire; HRQoL, health-related quality of life; OGC, oral glucocorticoid; WPAI, Work Productivity and Activity Impairment.

A total of 177 patients completed the EQ-5D-5L. Mean (SD) age was 49.2 (14.2) years, and the distribution of females (51%, n = 91/177) and males (49%, n = 86/177) was similar to that reported for the overall population. The mean (SD) EQ-5D-5L score was 0.72 (0.24); an EQ-5D-5L score closer to 1 indicates better health, and a score of 0 indicates worst health. Patients in remission had a mean (SD) EQ-5D-5L score of 0.77 (0.20), whereas the scores for patients in relapse or with refractory disease were 0.64 (0.15) and 0.50 (0.32), respectively. EQ-5D-5L scores (mean [SD]) were also lower than the overall mean score for patients with: moderate disease (0.56 [0.22]), severe disease (0.27 [0.34]), deteriorating disease prognosis (0.52 [0.24]), ≥3 symptoms at survey completion (0.61 [0.25]), EGPA-related organ damage (0.69 [0.24]), and blood eosinophil (bEOS) count ≥300 cells/µL (0.67 [0.22]). Patients requiring caregiver support or receiving oral glucocorticoids ≥10 mg/day also had EQ-5D-5L scores lower than the mean (0.58 [0.23] and 0.57 [0.29], respectively) (Fig 5).

A total of 82 patients completed the WPAI questionnaire. Mean (SD) age was 46.0 (10.4) years, and there was a lower proportion of females (44%, n = 36/82) than males (56%, n = 46/82). Mean (SD) overall work impairment was 20.8% (21.9); scores closer to 0% indicate no impairment, and a score of 100% indicates maximum impairment. Patients in remission had a mean (SD) overall work impairment score of 17.7% (19.7), whereas the score for patients in relapse or with refractory disease was 38.0% (24.9) and 34.5% (28.7), respectively. Overall work impairment scores (mean [SD]) were higher than the overall mean score for patients with moderate disease (42.7% [19.4]), severe disease (49.2% [23.6]), deteriorating disease prognosis (50.6% [18.6]), and bEOS ≥300 cells/µL (30.7% [25.1]). Patients requiring caregiver support also had higher overall work impairment scores than the mean (mean [SD]: 41.3% [25.5]) (Fig 5).

The mean (SD) ACQ score for patients with EGPA (n = 175) was 1.4 (1.0); an ACQ score closer to 0 than 6 indicates less impairment.

### Physician vs patient perceptions

Physician-provided patient data were matched to the equivalent patient-provided data for 180 patients. Reporting of disease severity at diagnosis and survey completion tended to differ between physicians and matched patients. At diagnosis, physicians perceived severe disease for 26% (n = 47/180) of patients, whereas 41% (n = 73/180) of patients perceived they had severe disease. Although there was an increase in patients with no active disease at survey completion, this was perceived by a smaller proportion of patients (13%, n = 22/174) than physicians (26%, n = 47/180) (Supplementary Fig).

The list of symptoms that physicians and patients could choose from differed, as the physicians’ symptom list was based on the BVAS and included signs of disease too, making direct comparisons challenging. Fatigue was reported by 52% (n = 93/179) of patients but was not reported by physicians as it was not captured in their survey. Similarly, 38% (n = 68/179) of patients reported shortness of breath, but this was not reported by physicians, as it was not an option provided. There were discrepancies between patients and physicians in the reporting of signs/symptoms that were present on the PRF and PSC: wheeze was reported by 54% (n = 97/180) of physicians but by only 40% (n = 71/179) of patients, and hypertension/high blood pressure was reported by 13% (n = 24/180) of physicians but by only 8% (n = 14/179) of patients (Supplementary Table S7).

Only 16% (n = 22/137) of patients classified as being in remission by physicians reported no signs/symptoms. The most frequently reported signs/symptoms among patients classified as being in remission were fatigue (50%, n = 68/137), wheezing (40%, n = 55/137), and shortness of breath (34%, n = 46/137).

### HCRU

Among the 401 patients surveyed, physicians reported a total of 139 emergency room visits due to EGPA in 86 patients during the 12 months prior to the survey. In addition, out of 406 patients surveyed, 72 were hospitalised for EGPA during the same period. The top reason for most recent hospitalisations was to treat a complication of EGPA (71%, n = 51/72). Of the patients who experienced an asthma exacerbation in the 12 months prior to survey completion, almost a quarter (24%, n = 20/83) were hospitalised due to their exacerbation (Supplementary Table S8).

## DISCUSSION

In this study, patients with EGPA faced a high disease burden, had moderate or severe disease at diagnosis, and experienced a wide range of clinical manifestations of EGPA.

Patients experienced a wait time of approximately 10 months from sign/symptom onset to EGPA diagnosis, during which time they typically had a high number of HCP encounters. This time from sign/symptom onset to diagnosis is less than that reported previously [[26], [27], [28]], which could be due to an increased awareness of EGPA owing to newer guidelines and the availability of approved therapies. Additionally, the current study selected consulting physicians who were more familiar with EGPA, as physicians were required to already be managing 2 or more patients with this condition. As a result, patients with early allergic signs and/or symptoms, such as asthma or nasal polyps, not meeting EGPA diagnostic criteria may have been missed if managed by other physicians, potentially leading to these patients not being captured in this data set; this may have led to the shorter time to diagnosis in our study, compared with other studies using insurance claims or other data sources [[26], [27], [28]]. Nevertheless, the maximum time from sign/symptom onset to diagnosis in this study was high (over 15 years), and patients had moderate-to-severe disease by the time of diagnosis. This underscores the need for increased disease awareness to facilitate prompt diagnosis and treatment.

In this study, patients had a high disease burden. Notably, there was discordance between physician and patient perspectives in terms of disease severity and signs and/or symptoms reported. A higher proportion of patients perceived their disease as severe at diagnosis and survey completion compared with physician assessments. Physicians more frequently reported wheeze and hypertension, whereas fatigue was the most commonly reported symptom among patients. To note, the symptom lists differed slightly between patients and physicians, with the latter receiving a list aligned with items from the BVAS, which also included signs of disease. This likely contributed to discrepancies in symptom reporting. Nevertheless, the discordance in physician and patient perspectives aligns with findings across multiple therapeutic areas [[29], [30], [31], [32]] and highlights how physicians may focus on measurable signs/symptoms and underestimate disease burden in terms of the impact on patients’ lives. This is further supported by the observation that a substantial proportion of patients classified as being in remission by physicians in this study nonetheless reported persistent symptoms.

Glucocorticoids were the predominant treatment prescribed for EGPA in this study, despite their associated negative effects. This is consistent with retrospective, longitudinal studies in the UK and Europe reporting ongoing reliance on oral glucocorticoid therapy for treatment of EGPA [2,3]. Overall, a low percentage of patients were receiving anti-IL-5/Rα treatments in this study, with the lowest prescriptions of these therapies from physicians in the UK, Germany, France, and the USA. At the time of study conduct, mepolizumab had been approved for treatment of EGPA in Europe for approximately 2 years and in the USA for approximately 6 years [12,13,33,34]. Additionally, at the time of study, mepolizumab was not reimbursed at the EGPA dose in the UK [35]. The relatively recent approval, particularly in Europe, and the lack of reimbursement in the UK, may have influenced prescribing patterns observed in this study. Despite recommendations for the use of anti-IL-5/Rα therapies for EGPA in rheumatology guidelines [4,9], these agents were infrequently prescribed by rheumatologists. Pulmonologists and allergists prescribed anti-IL-5/Rα therapies more frequently than other specialists, which may reflect earlier approval of these agents for the treatment of severe eosinophilic asthma and a greater familiarity with their use. In some cases, patients may have initially been prescribed these therapies under a diagnosis of severe eosinophilic asthma before the formal diagnosis of EGPA, particularly in healthcare settings where access to anti-IL-5/Rα therapies for EGPA was limited. Findings from this study suggest an overreliance on glucocorticoids despite their limitations and highlight the need for a greater awareness of the benefits of biologic agents, such as anti-IL-5/Rα, for reducing glucocorticoid dose and usage in patients with EGPA.

Patients in remission or who had no active disease in the current study had an HRQoL similar to that reported for the general population [36]. However, HRQoL and WPAI were notably impacted in patients with organ damage, an oral glucocorticoid dose ≥10 mg/day, bEOS count ≥300 cells/μL, or relapse, refractory, deteriorating, moderate, or severe disease. These findings underscore the need for multidisciplinary management and targeted therapies that achieve remission to enhance patients’ HRQoL, as well as the need to consider these outcomes in therapeutic trials.

A key strength of this study is that it provides real-world data from a large sample of patients with EGPA undergoing active HCP consultations for their disease, including pertinent patient-reported outcomes data. However, there were limitations. The study population was not a true random sample, which may limit generalisability. Physicians who were willing to participate were included, but they may not be reflective of all physicians treating EGPA. Moreover, patients were included based on the physician’s judgement rather than formal diagnostic or medical coding. Patient information was collected retrospectively, which may have introduced a recall bias, although that was mitigated by allowing physicians to consult medical records when completing the survey. In addition, patients and physicians selected symptoms from different predefined lists in their respective surveys, which limited the ability to make direct comparisons between patient- and physician-reported symptoms. In some cases, reported medications may have been prescribed for concomitant conditions other than EGPA, which may lead to misclassification of treatment patterns. Finally, data on HCRU were limited, as the PRF was not the most meticulous way to collect these data.

In summary, patients in this real-world study had a considerable disease burden by the time of diagnosis, highlighting the need for increased disease awareness to facilitate prompt diagnosis and treatment. There is a high reliance on glucocorticoids for EGPA treatment, and the use of biologics such as anti-IL-5/IL-5Rα therapies is low, highlighting a continuing need for the use of glucocorticoid-sparing, targeted therapies and optimised management to help patients achieve remission and enhance their HRQoL.

## Competing interests

The authors declare the following financial interests/relationships which may be considered as a potential conflict of interest: RS received: grants/research support from AbbVie, Amgen, AstraZeneca, Boehringer Ingelheim, Bristol Myers Squibb, Chemocentryx, Corbus, Cytori, GlaxoSmithKline, Kadmon, Novartis, and Roche-Genentech; and consulting fees from AbbVie, Amgen, AstraZeneca, Chemocentryx, Cytori, Galderma, GlaxoSmithKline, Novartis, Roche-Genentech, and Sanofi. PD, BD, PJ, LW, CE, and SYC are or were employees or contractors of AstraZeneca at the time of study conduct and may own stock/stock options. TP, OTC, and FH are or were employees of Adelphi Real World, which received funding from AstraZeneca to conduct this analysis.

## Appendix Supplementary materials

Image, application 1

Image, application 2

## Footnotes

Supplementary material associated with this article can be found, in the online version, at doi:10.1016/j.ero.2026.03.016.
