# Supplementary material for: Real-world disease burden, patient journey and treatment patterns in eosinophilic granulomatosis with polyangiitis in Europe and the USA
Source: EULAR Rheumatol Open. 2026 Apr 22;2(2):100168. doi: 10.1016/j.ero.2026.03.016 (PMC13425220; doi:10.1016/j.ero.2026.03.016)
Supplement: Supplementary file 2 [file mmc2.docx]

# Supplementary Materials

### Supplementary Table S1. Additional patient demographics and clinical characteristics at the time of survey

| **Variable** | **Total patients with EGPA  (N = 503)** |
| --- | --- |
| **Employment status, n (%)** | **n = 503** |
| Working full time | 234 (47) |
| Not working due to retirement | 83 (17) |
| Working part time | 65 (13) |
| Homemaker | 35 (7) |
| Student | 31 (6) |
| Unemployed | 29 (6) |
| On long term sick leave | 14 (3) |
| Don’t know | 12 (2) |
| **Working part time/not working due to retirement/unemployed/on long term sick leave, n (%)** | **n = 191** |
| Yes | 50 (26) |
| No | 119 (62) |
| Don’t know | 22 (12) |
| **Physician-perceived expected disease progression, n (%)^a^** | **n = 503** |
| Deteriorate rapidly | 6 (1) |
| Deteriorate moderately | 19 (4) |
| Deteriorate slowly | 44 (9) |
| Remain stable | 219 (44) |
| Improve slowly | 113 (22) |
| Improve moderately | 53 (11) |
| Improve rapidly | 27 (5) |
| Too early to determine | 22 (4) |
| **Most recent peak bEOS, n (%)** | **n = 288** |
| <300 cells/µL | 107 (37) |
| 300–499 cells/µL | 76 (26) |
| 500–999 cells/µL | 69 (24) |
| 1000–1499 cells/µL | 23 (8) |
| 1500–2499 cells/µL | 11 (4) |
| ≥2500 cells/µL | 2 (1) |

^a^No definitions of disease progression were provided in the survey, so the definitions of disease progression were based on each physician’s own judgement. bEOS, blood eosinophils; EGPA, eosinophilic granulomatosis with polyangiitis.

### Supplementary Table S2. Full patient journey information

| **Variable** | **Total patients with EGPA  (N = 503)** |
| --- | --- |
| **Time between sign/symptom onset and first consult (months)** | **n = 372** |
| Mean (SD); [min, max] | 3.8 (9.8); [0, 133.1] |
| **Time between first consult and EGPA diagnosis (months)** | **n = 385** |
| Mean (SD); [min, max] | 6.0 (14.5); [0, 142.1] |
| **Time between sign/symptom onset and EGPA diagnosis (months)** | **n = 376** |
| Mean (SD); [min, max] | 9.8 (19.1); [0, 183.3] |
| **Age at sign/symptom onset (years)** | **n = 390** |
| Mean (SD); [min, max] | 45.7 (14.6); [0, 88] |
| **Age at first consult for EGPA (years)** | **n = 389** |
| Mean (SD); [min, max] | 45.8 (14.6); [0, 88] |
| **Age at EGPA diagnosis (years)** | **n = 423** |
| Mean (SD); [min, max] | 46.5 (14.7); [10, 89] |
| **HCP who first consulted with patient, n (%)^a^** | **n = 503** |
| GP/PCP | 317 (63) |
| Emergency department physician | 34 (7) |
| Rheumatologist | 27 (5) |
| Pulmonologist | 24 (5) |
| Internist (Internal Medicine) | 17 (3) |
| Allergist/Immunologist | 14 (3) |
| Neurologist | 12 (2) |
| Gastroenterologist | 7 (1) |
| Dermatologist | 7 (1) |
| Haematologist | 6 (1) |
| Cardiologist | 5 (1) |
| Otolaryngologist | 3 (1) |
| Other HCP | 1 (<1) |
| Nephrologist | 1 (<1) |
| Don't know | 28 (6) |
| **HCP who reviewed the patient’s signs/symptoms, n (%)^b^** | **n = 503** |
| GP/PCP | 209 (42) |
| Pulmonologist | 181 (36) |
| Rheumatologist | 143 (28) |
| Internist (Internal Medicine) | 108 (21) |
| Allergist/Immunologist | 106 (21) |
| Gastroenterologist | 71 (14) |
| Otolaryngologist | 62 (12) |
| Dermatologist | 58 (12) |
| Emergency department physician | 54 (11) |
| Neurologist | 50 (10) |
| Cardiologist | 48 (10) |
| Haematologist | 47 (9) |
| Don't know | 25 (5) |
| Nephrologist | 23 (5) |
| Nurse | 7 (1) |
| Other HCP | 1 (<1) |
| None | 6 (1) |
| **HCP who diagnosed the patient’s EGPA, n (%)^a^** | **n = 503** |
| Rheumatologist | 120 (24) |
| Pulmonologist | 89 (18) |
| Internist (Internal Medicine) | 77 (15) |
| Allergist/Immunologist | 39 (8) |
| Haematologist | 31 (6) |
| Gastroenterologist | 28 (6) |
| Neurologist | 28 (6) |
| Dermatologist | 21 (4) |
| GP/PCP | 20 (4) |
| Cardiologist | 16 (3) |
| Emergency department physician | 5 (1) |
| Otolaryngologist | 4 (1) |
| Nephrologist | 4 (1) |
| Don't know | 21 (4) |
| **Total number of consultations prior to EGPA diagnosis** | **n = 120** |
| Mean (SD); [min, max] | 4.6 (4.0); [1, 23] |
| **HCP who initiated treatment, n (%)^a^** | **n = 503** |
| Rheumatologist | 140 (28) |
| Pulmonologist | 85 (17) |
| Internist (Internal Medicine) | 69 (14) |
| Allergist/Immunologist | 40 (8) |
| Haematologist | 36 (7) |
| Neurologist | 35 (7) |
| Gastroenterologist | 32 (6) |
| Dermatologist | 25 (5) |
| Cardiologist | 16 (3) |
| GP/PCP | 4 (1) |
| Nephrologist | 3 (1) |
| Otolaryngologist | 2 (<1) |
| Emergency department physician | 1 (<1) |
| Don't know | 15 (3) |
| **HCPs involved in treatment decisions the past 12 months,  n (%)^b^** | **n = 503** |
| Pulmonologist | 185 (37) |
| Rheumatologist | 172 (34) |
| Internist (Internal Medicine) | 88 (17) |
| GP/PCP | 79 (16) |
| Neurologist | 72 (14) |
| Gastroenterologist | 66 (13) |
| Dermatologist | 64 (13) |
| Allergist/Immunologist | 62 (12) |
| Haematologist | 58 (12) |
| Cardiologist | 57 (11) |
| Otolaryngologist | 44 (9) |
| Nephrologist | 18 (4) |
| Nurse | 11 (2) |
| Emergency department physician | 7 (1) |
| **When HCP started to manage patient, n (%)** | **n = 423** |
| Managed **on** same day of diagnosis | 192 (45) |
| Managed **after** patient was diagnosed | 163 (39) |
| Managed **before** patient was diagnosed | 68 (16) |
| **Total number of signs and/or symptoms experienced at diagnosis** | **n = 503** |
| Mean (SD); [min, max] | 5.5 (3.8); [1, 25] |
| **bEOS (at diagnosis), n (%)** | **n = 503** |
| <300 cells/µL | 6 (1) |
| 300–499 cells/µL | 19 (4) |
| 500–999 cells/µL | 60 (12) |
| 1000–1499 cells/µL | 150 (30) |
| 1500–2499 cells/µL | 149 (30) |
| ≥2500 cells/µL | 93 (18) |
| The patient’s peak bEOS was collected, but did not have a record of the score | 21 (4) |
| Did not collect bEOS at diagnosis | 5 (1) |
| **Physician-perceived severity of EGPA at diagnosis, n (%)** | **n = 503** |
| Mild | 99 (20) |
| Moderate | 277 (55) |
| Severe | 122 (24) |
| Don’t know | 5 (1) |

^a^Physicians could select a single response. ^b^Physicians could select multiple responses. ^c^No definitions of mild, moderate or severe were provided in the survey, so the definitions of severity were based on each physician’s own judgement. bEOS, blood eosinophils; EGPA, eosinophilic granulomatosis with polyangiitis; GP, general practitioner; HCP, healthcare professional; PCP, primary-care physician; SD, standard deviation.

### Supplementary Table S3. Physician-reported signs and symptoms experienced by patients at diagnosis and survey completion

| **Signs and symptoms experienced  by patients** | **At  diagnosis (n = 503)** | **At survey completion  (n = 503)** | **Signs and symptoms experienced  by patients** | **At  diagnosis (n = 503)** | **At survey completion  (n = 503)** |
| --- | --- | --- | --- | --- | --- |
| Wheeze | 261 (52%) | 185 (37%) | Blurred vision | 24 (5%) | 8 (2%) |
| Purpura | 195 (39%) | 99 (20%) | Massive hemoptysis/ alveolar hemorrhage | 24 (5%) | 4 (1%) |
| Paranasal sinus involvement | 183 (36%) | 128 (25%) | Uveitis | 21 (4%) | 12 (2%) |
| Myalgia | 152 (30%) | 87 (17%) | Infarct | 20 (4%) | 9 (2%) |
| Infiltrate | 151 (30%) | 61 (12%) | Conductive hearing loss | 20 (4%) | 18 (4%) |
| Arthralgia/arthritis | 124 (25%) | 83 (17%) | Subglottic stenosis | 19 (4%) | 7 (1%) |
| Skin ulcer | 100 (20%) | 37 (7%) | Ischaemic cardiac pain | 19 (4%) | 17 (3%) |
| Weight loss ≥2 kg | 95 (19%) | 13 (3%) | Genital ulcers | 17 (3%) | 6 (1%) |
| Pleural effusion/ pleurisy | 92 (18%) | 28 (6%) | Adnexal inflammation | 14 (3%) | 5 (1%) |
| Fever ≥38°C | 90 (18%) | 11 (2%) | Creatinine 250–499 µmol/L (2.83–5.65 mg/dL) | 13 (3%) | 5 (1%) |
| Bloody nasal discharge/crusts/ ulcer/granulomata | 89 (18%) | 37 (7%) | Peritonitis | 12 (2%) | 3 (1%) |
| Sensory peripheral neuropathy | 72 (14%) | 66 (13%) | Organic confusion | 12 (2%) | 4 (1%) |
| Hypertension | 70 (14%) | 64 (13%) | Loss of pulse | 11 (2%) | 7 (1%) |
| Other skin vasculitis | 69 (14%) | 34 (7%) | Cerebrovascular accident | 11 (2%) | 5 (1%) |
| Headache | 68 (14%) | 45 (9%) | Significant proptosis | 10 (2%) | 2 (<1%) |
| Nodules or cavities | 68 (14%) | 28 (6%) | Gangrene | 9 (2%) | 4 (1%) |
| Ischaemic abdominal pain | 61 (12%) | 37 (7%) | Valvular heart disease | 9 (2%) | 8 (2%) |
| Mouth ulcers | 56 (11%) | 24 (5%) | Cranial nerve palsy | 9 (2%) | 6 (1%) |
| Pericarditis | 54 (11%) | 18 (4%) | Sensorineural hearing loss | 8 (2%) | 7 (1%) |
| Bloody diarrhoea | 54 (11%) | 36 (7%) | Congestive heart failure | 8 (2%) | 6 (1%) |
| Proteinuria >1 g/day | 53 (11%) | 24 (5%) | Retinal changes (vasculitis/thrombosis/ exudate/ haemorrhage) | 8 (2%) | 3 (1%) |
| Conjunctivitis/ blepharitis/keratitis | 49 (10%) | 26 (5%) | Seizure (not hypertensive) | 8 (2%) | 4 (1%) |
| Haematuria ≥10 red blood cells/high power field | 41 (8%) | 15 (3%) | Other | 8 (2%) | 11 (2%) |
| Creatinine 125–249 µmol/L (1.41–2.82 mg/dL) | 39 (8%) | 36 (7%) | Spinal cord lesion | 6 (1%) | 4 (1%) |
| Endobronchial involvement | 38 (8%) | 18 (4%) | Sudden vision loss | 4 (1%) | 2 (<1%) |
| Mononeuritis multiplex | 34 (7%) | 24 (5%) | Rise in serum creatinine <30%, or fall in creatinine clearance >25% | 3 (1%) | 1 (<1%) |
| Cardiomyopathy | 31 (6%) | 22 (4%) | Creatinine ≥500 µmol/L (≥5.66 mg/dL) | 1 (<1%) | 1 (<1%) |
| Respiratory failure | 27 (5%) | 11 (2%) | Meningitis | - | 1 (<1%) |
| Scleritis/episcleritis | 26 (5%) | 16 (3%) |  |  |  |

### Supplementary Table S4. Full list of concomitant conditions

| **Concomitant conditions, n (%)** | **Patients with EGPA (N = 503)** |
| --- | --- |
| Asthma | 277 (55) |
| Allergic rhinitis | 109 (22) |
| Chronic rhinosinusitis | 93 (19) |
| Nasal polyps | 71 (14) |
| Pulmonary infiltrates | 68 (14) |
| Chronic pulmonary disease | 64 (13) |
| Vasculitis | 61 (12) |
| Atopic dermatitis | 59 (12) |
| Renal disease | 35 (7) |
| Peptic ulcer disease | 34 (7) |
| Congestive heart failure | 32 (6) |
| Rheumatologic disease | 31 (6) |
| Polyneuropathy | 30 (6) |
| Peripheral vascular disease | 27 (5) |
| Chronic obstructive pulmonary disease | 26 (5) |
| Eosinophilic esophagitis | 23 (5) |
| Multiple mononeuropathy | 21 (4) |
| Myocarditis | 19 (4) |
| Hand and/or foot eczema | 19 (4) |
| Other (not already listed here, or previously) | 18 (4) |
| Mild liver disease | 18 (4) |
| Diabetes without chronic complications | 16 (3) |
| Chronic spontaneous urticaria | 14 (3) |
| Long term effects of COVID-19 | 13 (3) |
| Other eosinophilic gastrointestinal disease | 11 (2) |
| Inflammatory bowel disease | 10 (2) |
| Myocardial infarction | 10 (2) |
| Diabetes with chronic complications | 8 (2) |
| Psoriatic arthritis | 8 (2) |
| Cerebrovascular disease | 7 (1) |
| Psoriasis | 6 (1) |
| Crohn’s disease | 5 (1) |
| Hemiplegia or paraplegia | 4 (1) |
| Metastatic solid | 3 (1) |
| Moderate or severe liver disease | 2 (<1) |
| Dementia | 2 (<1) |
| Ulcerative colitis | 1 (<1) |
| AIDS/HIV | 1 (<1) |
| Any malignancy, including lymphoma | 1 (<1) |
| Not diagnosed with any concomitant conditions | 184 (37) |

AIDS, acquired immunodeficiency syndrome; EGPA, eosinophilic granulomatosis with polyangiitis; HIV, human immunodeficiency virus.

### Supplementary Table S5. Additional information on treatment patterns

| **Variable** | **Total patients with EGPA  (N = 503)** |
| --- | --- |
| **Type of treatment, n (%)** | **n = 499** |
| Maintain remission | 529 (106) |
| Induce remission | 352 (71) |
| Bridging therapy | 48 (10) |
| Another reason for prescription | 33 (7) |
| **Months on current pharmacological treatment** | **n = 499** |
| Mean (SD); [min, max] | 19.7 (27.7); [0, 226] |
| **Glucocorticoid^a^ currently prescribed, n (%)** | **n = 351** |
| Prednisone | 213 (61) |
| Prednisolone | 94 (27) |
| Methylprednisolone | 36 (10) |
| Dexamethasone | 6 (2) |
| Betamethasone | 2 (1) |
| **Glucocorticoid^a^ dosage (mg/day, prednisone equivalent)** | **n = 351** |
| Mean | 17.0 |
| Min | 1 |
| Median | 8.0 |
| Max | 120 |
| SD | 20.4 |
| **Glucocorticoid^a^ current dosage (grouped) (mg/day, prednisone equivalent) n (%)** | **n = 351** |
| ≤4 | 36 (10) |
| 4.1–7.5 | 131 (37) |
| 7.6–10 | 57 (16) |
| 11–20 | 55 (16) |
| 21–30 | 16 (5) |
| 31–40 | 19 (5) |
| >40 | 37 (11) |
| **Dosing regimen for mepolizumab, n (%)** | **n = 105** |
| 100 mg every 4 weeks | 16 (15) |
| 300 mg every 4 weeks | 89 (85) |
| **Dosing regimen for rituximab, n (%)** | **n = 81** |
| 375 mg once a week for 4 weeks – followed by 500 mg every 6 months | 69 (85) |
| Other dosing regimen | 12 (15) |
| **Dosing regimen for benralizumab, n (%)** | **n = 33** |
| 30 mg every 4 weeks | 13 (39) |
| 30 mg – first 3 doses every 4 weeks, then every 8 weeks | 20 (61) |
| **Dosing regimen for omalizumab, n (%)** | **n = 21** |
| 150 mg every 4 weeks | 13 (62) |
| 300 mg every 4 weeks | 8 (38) |
| **Dosing regimen for dupilumab, n (%)** | **n = 15** |
| 300 mg every other week | 10 (67) |
| 400 mg once – followed by 200 mg every other week | 2 (13) |
| 600 mg once – followed by 300 mg every other week | 3 (20) |
| **Dosing regimen for reslizumab, n (%)** | **n = 2** |
| 3 mg/kg IV every 4 weeks | 2 (100) |
| **Physician-perceived severity of EGPA at start of treatment,  n (%)^b^** | **n = 499** |
| Mild | 125 (25) |
| Moderate | 265 (53) |
| Severe | 82 (16) |
| No active disease | 17 (3) |
| Don’t know | 10 (2) |
| **Physician satisfaction with current control of EGPA, n (%)** | **n = 489** |
| Yes, and I believe that this is the best control that can be realistically achieved for this patient | 264 (54) |
| Yes, but I believe better control can be achieved for this patient | 164 (34) |
| No, and I believe better control can be achieved for this patient | 25 (5) |
| No, but I believe this is the best control that can be realistically achieved for this patient | 36 (7) |
| **How patient receives their current treatment for EGPA, n (%)^d^** | **n = 499** |
| Patient self-administers at home (e.g. oral/topical treatments) | 315 (63) |
| Patient attends an appointment where the physician administers an injection/infusion | 197 (39) |
| **HCP who initiated current treatment, n (%)^c^** | **n = 499** |
| Rheumatologist | 95 (19) |
| Pulmonologist | 74 (15) |
| Gastroenterologist | 67 (13) |
| Internist (Internal Medicine) | 66 (13) |
| Allergist/Immunologist | 63 (13) |
| Haematologist | 40 (8) |
| Neurologist | 36 (7) |
| Dermatologist | 27 (5) |
| Cardiologist | 16 (3) |
| GP/PCP | 6 (1) |
| Otolaryngologist | 6 (1) |
| Nephrologist | 3 (1) |
| **Concomitant conditions a factor in prescribing a biologic,  n (%)**  *Only asked to patients with concomitant conditions* | **n = 171** |
| Yes | 99 (58) |
| No | 69 (40) |
| Don’t know | 3 (2) |
| **Biological treatment prescribed for concomitant conditions,  n (%)**  *Patients not receiving a biologic for their EGPA* | **n = 115** |
| Yes | 5 (4) |
| No | 102 (89) |
| Don’t know | 8 (7) |
| **Symptomatic improvement since starting current treatment,  n (%)** | **n = 499** |
| Yes, symptomatic severity has improved | 418 (84) |
| No improvement | 32 (6) |
| Patient’s condition has worsened since starting treatment | 6 (1) |
| Too early to tell | 43 (9) |
| **Signs and symptoms that have shown improvement, n (%)^d^** | **n = 418** |
| Wheeze | 205 (49) |
| Purpura | 142 (34) |
| Paranasal sinus involvement | 124 (30) |
| Infiltrate | 106 (25) |
| Myalgia | 81 (19) |
| Arthralgia/arthritis | 73 (17) |
| Skin ulcer | 65 (16) |
| Fever ≥38°C | 58 (14) |
| Bloody nasal charge/crusts/ulcer/granulomata | 57 (14) |
| Pleural effusion/pleurisy | 54 (13) |
| Other skin vasculitis | 46 (11) |
| Weight loss ≥2 kg | 46 (11) |
| Nodules or cavities | 43 (10) |
| Ischaemic abdominal pain | 41 (10) |
| Mouth ulcers | 36 (9) |
| Bloody diarrhoea | 34 (8) |
| Proteinuria >1 g/day | 34 (8) |
| Sensory peripheral neuropathy | 32 (8) |
| Pericarditis | 29 (7) |
| Headache | 28 (7) |
| Conjunctivitis/blepharitis/keratitis | 24 (6) |
| Creatinine 125–249 µmol/L (1.41–2.82 mg/dL) | 24 (6) |
| Endobronchial involvement | 22 (5) |
| Mononeuritis multiplex | 22 (5) |
| Respiratory failure | 20 (5) |
| Haematuria ≥10 red blood cells/high power field | 20 (5) |
| Hypertension | 17 (4) |
| Cardiomyopathy | 15 (4) |
| Blurred vision | 12 (3) |
| Scleritis/episcleritis | 12 (3) |
| Subglottic stenosis | 11 (3) |
| Uveitis | 11 (3) |
| Conductive hearing loss | 9 (2) |
| Massive haemoptysis/alveolar haemorrhage | 9 (2) |
| Infarct | 8 (2) |
| Genital ulcers | 8 (2) |
| Adnexal inflammation | 7 (2) |
| Creatinine 250–499 µmol/L (2.83–5.65 mg/dL) | 6 (1) |
| Sensorineural hearing loss | 6 (1) |
| Cerebrovascular accident | 6 (1) |
| Seizure (not hypertensive) | 5 (1) |
| Peritonitis | 5 (1) |
| Ischaemic cardiac pain | 5 (1) |
| Gangrene | 5 (1) |
| Cranial nerve palsy | 4 (1) |
| Congestive heart failure | 4 (1) |
| Retinal changes (vasculitis/thrombosis/exudate/haemorrhage) | 4 (1) |
| Organic confusion | 4 (1) |
| Significant proptosis | 4 (1) |
| Loss of pulse | 4 (1) |
| Spinal cord lesion | 4 (1) |
| Other | 3 (1) |
| Valvular heart disease | 2 (<1) |
| Rise in serum creatinine <30%, or fall in creatinine clearance >25% | 1 (<1) |
| Sudden vision loss | 1 (<1) |
| **Reasons for prescribing current treatment, n (%)^d^** | **n = 499** |
| Symptom relief | 306 (61) |
| Speed of onset of action | 287 (58) |
| Reduce current morbidity and organ damage | 215 (43) |
| Improves patient’s quality of life | 209 (42) |
| Prevent further morbidity and organ damage | 198 (40) |
| Reduce bEOS/remission | 185 (37) |
| Reduce mortality | 180 (36) |
| Well tolerated side-effect profile | 149 (30) |
| Reduce dose and/or duration of steroids (steroid sparing) | 149 (30) |
| Provides an improvement in a concomitant condition (e.g. asthma and/or atopic dermatitis) | 123 (25) |
| Drug familiarity and personal experience | 101 (20) |
| Ease of use and administration | 99 (20) |
| In accordance with clinical guidelines | 99 (20) |
| Reduction in use of other background medications | 61 (12) |
| Increases patient compliance | 57 (11) |
| Reduces the need for prescribing biologics | 51 (10) |
| Available on formulary | 45 (9) |
| Effective treatment plan at a low cost for the patient | 35 (7) |
| Insurance coverage | 30 (6) |
| Reduction in frequency of physician consultations | 20 (4) |
| Requested by patient | 12 (2) |
| Effective treatment plan at a low cost to my/the practice | 10 (2) |
| **Areas for improvement, n (%)^d^** | **n = 499** |
| Speed of onset of action | 132 (26) |
| Well tolerated side-effect profile | 130 (26) |
| Reduce mortality | 85 (17) |
| Prevent further morbidity and organ damage | 79 (16) |
| Reduce dose and/or duration of steroids (steroid sparing) | 73 (15) |
| Reduce current morbidity and organ damage | 72 (14) |
| Symptom relief | 68 (14) |
| Improves patient’s quality of life | 56 (11) |
| Reduce bEOS count/remission | 42 (8) |
| Reduces the need for prescribing biologics | 37 (7) |
| Ease of use and administration | 35 (7) |
| Increases patient compliance | 31 (6) |
| Provides an improvement in a concomitant condition (e.g. asthma and/or atopic dermatitis) | 30 (6) |
| Reduction in use of other background medications | 30 (6) |
| Reduction in frequency of physician consultations | 26 (5) |
| Other | 1 (<1) |

^a^Oral and/or parenteral glucocorticoids; ^b^No definitions of mild, moderate or severe were provided in the survey, so the definitions of severity were based on each physician’s own judgement; ^c^Physicians could select a single response; ^d^Physicians could select multiple responses. bEOS, blood eosinophils; EGPA, eosinophilic granulomatosis with polyangiitis; GP, general practitioner; HCP, healthcare professional; IV, intravenous; PCP, primary-care physician; SD, standard deviation.

### Supplementary Table S6. Pharmacological treatments currently prescribed, stratified by country

| **Treatment** | **Country/region** | | | | | | | |
| --- | --- | --- | --- | --- | --- | --- | --- | --- |
|  | **Total n = 503** | **France n = 80** | **Germany n = 80** | **Italy n = 91** | **Spain n = 75** | **UK n = 57** | **EU5 n = 383** | **US n = 120** |
| **Any glucocorticoid, n (%)** | **399 (79)** | **64 (80)** | **65 (81)** | **73 (80)** | **60 (80)** | **49 (86)** | **311 (81)** | **88 (73)** |
| Oral glucocorticoid | 338 (67) | 52 (65) | 58 (73) | 61 (67) | 48 (64) | 45 (79) | 264 (69) | 74 (62) |
| Inhaled/nasal glucocorticoid | 112 (22) | 22 (28) | 19 (24) | 24 (26) | 22 (29) | 6 (11) | 93 (24) | 19 (16) |
| Parenteral glucocorticoid | 26 (5) | 8 (10) | 1 (1) | 8 (9) | 2 (3) | 6 (11) | 25 (7) | 1 (1) |
| Topical glucocorticoid | 26 (5) | - | 3 (4) | 8 (9) | 3 (4) | 4 (7) | 18 (5) | 8 (7) |
| **Any biologic, n (%)** | **251 (50)** | **27 (34)** | **29 (36)** | **68 (75)** | **51 (68)** | **23 (40)** | **198 (52)** | **53 (44)** |
| Mepolizumab | 105 (21) | 13 (16) | 17 (21) | 38 (42) | 17 (23) | 6 (11) | 91 (24) | 14 (12) |
| Benralizumab | 33 (7) | 1 (1) | - | 8 (9) | 8 (11) | 10 (18) | 27 (7) | 6 (5) |
| Omalizumab | 21 (4) | 1 (1) | 5 (6) | - | 6 (8) | 2 (4) | 14 (4) | 7 (6) |
| Dupilumab | 15 (3) | 3 (4) | - | 1 (1) | 2 (3) | - | 6 (2) | 9 (8) |
| Reslizumab | 2 (<1) | - | - | - | 1 (1) | - | 1 (<1) | 1 (1) |
| Rituximab | 81 (16) | 10 (13) | 10 (13) | 21 (23) | 17 (23) | 6 (11) | 64 (17) | 17 (14) |
| **Any immunosuppressant, n (%)** | **183 (36)** | **27 (34)** | **32 (40)** | **41 (45)** | **31 (41)** | **22 (39)** | **153 (40)** | **30 (25)** |
| Methotrexate | 63 (13) | 7 (9) | 18 (23) | 10 (11) | 7 (9) | 8 (14) | 50 (13) | 13 (11) |
| Azathioprine | 59 (12) | 12 (15) | 6 (8) | 16 (18) | 8 (11) | 7 (12) | 49 (13) | 10 (8) |
| Mycophenolate mofetil | 43 (9) | 4 (5) | 2 (3) | 11 (12) | 15 (20) | 4 (7) | 36 (9) | 7 (6) |
| Cyclophosphamide | 25 (5) | 6 (8) | 7 (9) | 4 (4) | 1 (1) | 4 (7) | 22 (6) | 3 (3) |
| **IVIg/plasmapheresis/PLEX, n (%)** | **10 (2)** | **-** | **-** | **1 (1)** | **-** | **2 (4)** | **3 (1)** | **7 (6)** |
| IVIg | 9 (2) | - | - | 1 (1) | - | 2 (4) | 3 (1) | 6 (5) |
| PLEX | 1 (<1) | - | - | - | - | - | - | 1 (1) |
| Other treatment | 3 (1) | 1 (1) | - | - | 2 (3) | - | 3 (1) | - |
| **None, n (%)** | **4 (1)** | **2 (3)** | **1 (1)** | **-** | **-** | **-** | **3 (1)** | **1 (1)** |

IVIg, intravenous immunoglobin; PLEX, plasma exchange.

### Supplementary Table S7. Reporting of signs and symptoms from matched physicians and patients at survey completion

| **Physician responses: which of the following signs/symptoms is this patient currently experiencing?** **(based off BVAS version 3)** | | **Patient responses: which of the following symptoms have you experienced in the last 4 weeks?** | |
| --- | --- | --- | --- |
| **Base** | **n = 180** | **Base** | **n = 179** |
| Wheeze | 97 (54%) | Fatigue/tiredness | 93 (52%) |
| Paranasal sinus involvement | 53 (29%) | Wheezing | 71 (40%) |
| Arthralgia/arthritis | 49 (27%) | Shortness of breath | 68 (38%) |
| Myalgia | 48 (27%) | Joint pain | 65 (36%) |
| Purpura | 32 (18%) | Muscle pain | 60 (34%) |
| Sensory peripheral neuropathy | 31 (17%) | Persistent cough | 46 (26%) |
| Headache | 28 (16%) | Skin rashes/itchiness | 42 (23%) |
| Infiltrate | 25 (14%) | Trouble breathing (general) | 37 (21%) |
| Hypertension | 24 (13%) | Blocked sinuses | 36 (20%) |
| Ischaemic abdominal pain | 14 (8%) | Headache | 36 (20%) |
| Other skin vasculitis | 13 (7%) | Difficulty sleeping | 34 (19%) |
| Creatinine 125–249 µmol/L (1.41–2.82 mg/dl) | 13 (7%) | Skin discolouration | 33 (18%) |
| Skin ulcer | 12 (7%) | Skin sensitivity/pain | 32 (18%) |
| Bloody nasal discharge/crusts/ ulcer/granulomata | 12 (7%) | Joint stiffness | 31 (17%) |
| Mononeuritis multiplex | 10 (6%) | Pins and needles in hands and/or feet | 30 (17%) |
| Nodules or cavities | 10 (6%) | Numbness in hands and/or feet | 28 (16%) |
| Pleural effusion/pleurisy | 10 (6%) | Increased discharge from nose | 28 (16%) |
| Conjunctivitis/blepharitis/keratitis | 9 (5%) | Abdominal pain | 25 (14%) |
| Endobronchial involvement | 9 (5%) | Anxiety | 22 (12%) |
| Conductive hearing loss | 8 (4%) | Trouble breathing (through nose) | 19 (11%) |
| Mouth ulcers | 8 (4%) | Feeling bloated | 18 (10%) |
| Scleritis/Episcleritis | 8 (4%) | Skin ulcer/hives | 18 (10%) |
| Ischaemic cardiac pain | 8 (4%) | Loss of appetite | 18 (10%) |
| Weight loss ≥2 kg | 8 (4%) | Loss of strength in hands and/or feet | 17 (9%) |
| Respiratory failure | 6 (3%) | Heartburn | 17 (9%) |
| Uveitis | 6 (3%) | Diarrhoea | 17 (9%) |
| Cardiomyopathy | 6 (3%) | Weight loss | 15 (8%) |
| Bloody diarrhoea | 4 (2%) | Burning sensation in hands and/or feet | 14 (8%) |
| Haematuria ≥10 red blood cells/high power field | 4 (2%) | High blood pressure | 14 (8%) |
| Blurred vision | 4 (2%) | Irritation/redness in eye(s) | 13 (7%) |
| Pericarditis | 3 (2%) | Dizziness | 12 (7%) |
| Subglottic stenosis | 3 (2%) | Chest pain | 12 (7%) |
| Valvular heart disease | 3 (2%) | Skin thickening/hardening | 10 (6%) |
| Fever ≥ 38°C | 3 (2%) | Ear fullness/blockage | 10 (6%) |
| Proteinuria >1 g/day | 3 (2%) | Pain in ears | 9 (5%) |
| Other | 3 (2%) | Fever | 9 (5%) |
| Sudden vision loss | 2 (1%) | Chills/sweats | 9 (5%) |
| Adnexal inflammation | 2 (1%) | Feeling sick/vomiting | 9 (5%) |
| Retinal changes (vasculitis/ thrombosis/exudate/haemorrhage) | 2 (1%) | Swelling of eye(s) | 9 (5%) |
| Sensorineural hearing loss | 2 (1%) | Loss of sense of smell | 8 (4%) |
| Peritonitis | 1 (1%) | Forgetfulness/confusion | 7 (4%) |
| Organic confusion | 1 (1%) | Pain in eye | 7 (4%) |
| Seizure (not hypertensive) | 1 (1%) | Hair loss | 7 (4%) |
| Cerebrovascular accident | 1 (1%) | Presence of blood in stools | 6 (3%) |
| Spinal cord lesion | 1 (1%) | Blurred vision | 6 (3%) |
| Loss of pulse | 1 (1%) | Difficulties with coordination | 6 (3%) |
| Significant proptosis | 1 (1%) | Mouth ulcers | 5 (3%) |
| Gangrene | 1 (1%) | Fast heartbeat | 5 (3%) |
| Massive haemoptysis/alveolar haemorrhage | 1 (1%) | Loss of hearing | 4 (2%) |
| Genital ulcers | 1 (1%) | Redness in/darker/red urine | 4 (2%) |
| Congestive heart failure | 1 (1%) | Heart pain | 4 (2%) |
| Creatinine 250–499 µmol/L (2.83–5.65 mg/dl) | 1 (1%) | Vision loss | 3 (2%) |
|  | | Facial pain | 3 (2%) |
|  |  | Weight gain | 2 (1%) |
|  |  | Genital ulcers/inflammation | 1 (1%) |
|  |  | Other symptom(s) | 1 (1%) |
|  |  | Seizures | 1 (1%) |
|  |  | No symptoms | 23 (13%) |

BVAS, Birmingham Vasculitis Activity Score.

### Supplementary Table S8. Healthcare resource utilisation

| **Variable** | **Total patients with EGPA  (N = 503)** |
| --- | --- |
| **Patients surveyed on number of visits to the ER (past 12 months), n (%)** | **n = 401** |
| Mean (SD); [min, max] | 0.3 (0.8); [0, 7] |
| 0 visits | 315 (78) |
| 1 visit | 51 (13) |
| 2 visits | 27 (7) |
| ≥3 visits | 8 (2) |
| Sum of visits to the ER due to EGPA | 139 |
| **Patients surveyed on number of hospitalisations (past 12 month), n (%)** | **n = 406** |
| Mean (SD); [min, max] | 0.2 (0.5); [0, 3] |
| 0 hospitalisations | 334 (82) |
| ≥1 hospitalisation(s) | 72 (18) |
| **Reason for hospitalisation, n (%)** | **n = 72** |
| To treat a complication of EGPA | 51 (71) |
| Other | 10 (14) |
| For surgery | 6 (8) |
| To manage an adverse treatment reaction | 4 (6) |
| Don’t know | 1 (1) |
| **Asthma exacerbation in the past 12 months, n (%)** | **n = 277** |
| Yes | 83 (30) |
| No | 194 (70) |
| **Hospitalisation due to an asthma exacerbation (past 12 months), n (%)** | **n = 83** |
| Yes | 20 (24) |
| No | 63 (76) |
| **Number of consults with a Pulmonologist (past 12 months)** | **n = 100** |
| Mean (SD); [min, max]^a^ | 3.0 (4.2); [1, 40] |
| **Number of consults with a GP/PCP (past 12 months)** | **n = 78** |
| Mean (SD); [min, max]^a^ | 4.0 (2.9); [1, 12] |
| **Number of consults with a Rheumatologist (past 12 months)** | **n = 69** |
| Mean (SD); [min, max]^a^ | 2.3 (1.8); [1, 11] |
| **Number of consults with a** **Cardiologist (past 12 months)** | **n = 43** |
| Mean (SD); [min, max]^a^ | 1.9 (1.2); [1, 5] |
| **Number of consults with an Otolaryngologist (past 12 months)** | **n = 40** |
| Mean (SD); [min, max]^a^ | 1.8 (1.0); [1, 4] |
| **Number of consults with an Internist (past 12 months)** | **n = 38** |
| Mean (SD); [min, max]^a^ | 3.6 (2.2); [1, 10] |
| **Number of consults with an Allergist/Immunologist (past 12 months)** | **n = 35** |
| Mean (SD); [min, max]^a^ | 2.7 (1.5); [1, 6] |
| **Number of consults with a Neurologist (past 12 months)** | **n = 31** |
| Mean (SD); [min, max]^a^ | 1.9 (1.1); [1, 4] |
| **Number of consults with a Dermatologist (past 12 months)** | **n = 29** |
| Mean (SD); [min, max]^a^ | 2.1 (1.2); [1, 7] |
| **Number of consults with a** **Gastroenterologist (past 12 months)** | **n = 23** |
| Mean (SD); [min, max]^a^ | 2.7 (2.0); [1, 7] |
| **Number of consults with a Nephrologist (past 12 months)** | **n = 18** |
| Mean (SD); [min, max]^a^ | 3.1 (2.0); [1, 10] |
| **Number of consults with a Haematologist (past 12 months)** | **n = 16** |
| Mean (SD); [min, max]^a^ | 5.1 (9.4); [1, 40] |
| **Number of consults with a Nurse (past 12 months)** | **n = 11** |
| Mean (SD); [min, max]^a^ | 4.5 (2.8); [2, 10] |
| **Number of consults with an Emergency Department physician (past 12 months)** | **n = 7** |
| Mean (SD); [min, max]^a^ | 2.1 (1.9); [1, 6] |

^a^Numbers only for those patients who consulted with the specific HCP. EGPA, eosinophilic granulomatosis with polyangiitis; ER, emergency room; GP, general practitioner; HCP, healthcare professional; PCP, primary-care physician; SD, standard deviation.

*
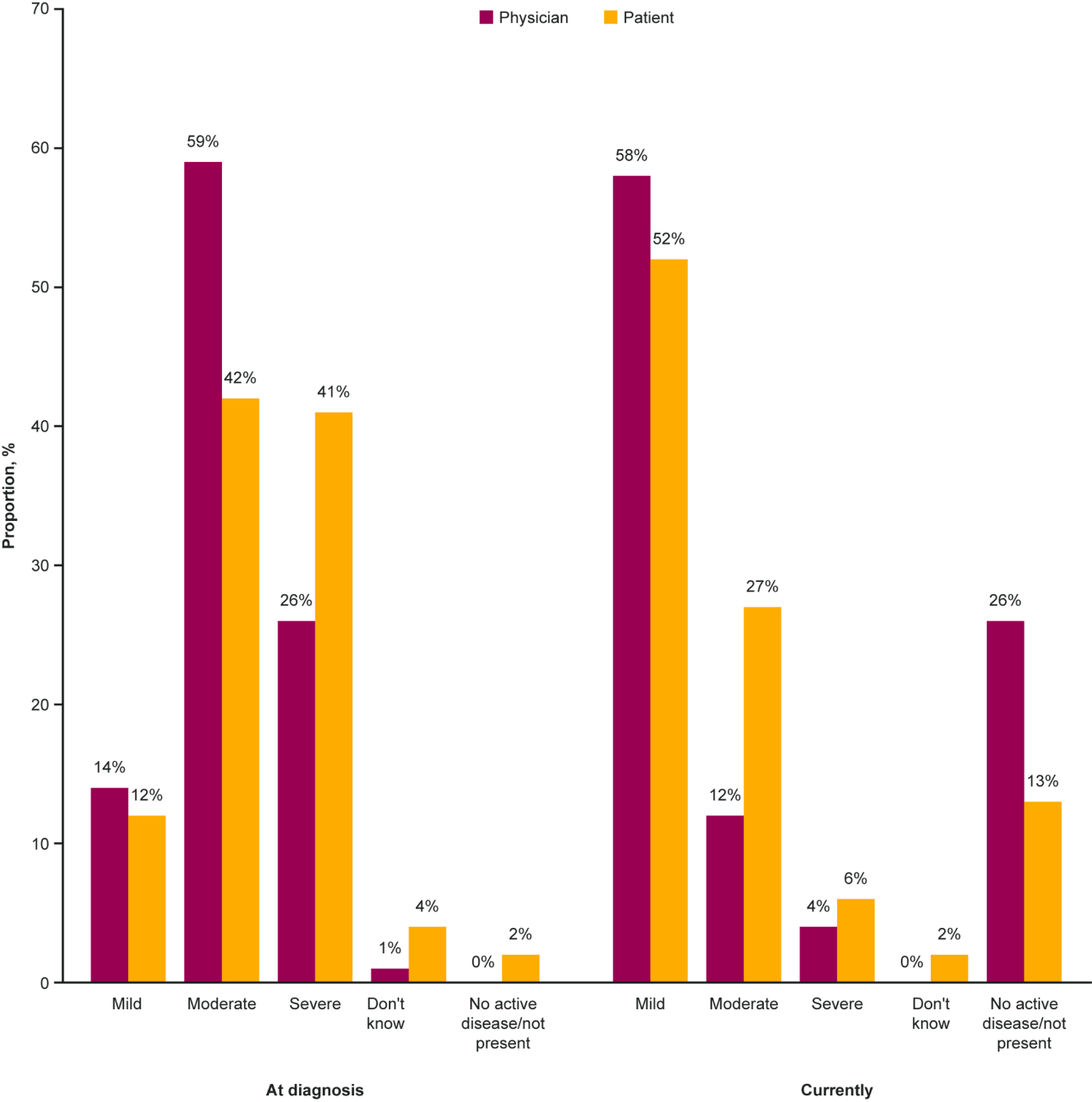
*

### Supplementary Figure. Matched physician- and patient-reported data on disease severity at diagnosis and survey completion.
